# Recognizing Egyptian currency for people with visual impairment using deep learning models

**DOI:** 10.1038/s41598-025-20646-x

**Published:** 2025-10-01

**Authors:** Ahmed M. Ghanem, Hassan A. Youness, Mohamed Wahba, Hammam M. Abdelaal

**Affiliations:** 1 Department of Computers & Systems Engineering, Faculty of Engineering, Minia University, Minya, 61519 Egypt; 2 Technology Center, Kafrelsheikh University, Kafr el-Sheikh, 33516 Egypt; 3 Earth Observation Data Management, Egyptian Space Agency, New Cairo, 11835 Egypt; 4 Department of Information Technology, Faculty of Computers and Information, Luxor University, Luxor, 85951 Egypt

**Keywords:** Artificial intelligence, YOLO, Generalized efficient layer aggregation network (GELAN), Neural network, Non-maximum suppression (NMS), Risk factors, Engineering, Mathematics and computing

## Abstract

This study presents a novel real-time Egyptian currency recognition system designed to assist visually impaired individuals in performing financial transactions independently and securely. The system leverages advanced deep learning models—YOLOv8, YOLOv9, and YOLOv10 to achieve high accuracy and low latency in identifying Egyptian banknotes. Evaluated on a comprehensive dataset of 2,000 annotated images, the models incorporate innovations such as context aggregation, GELAN, and NMS-free training to enhance performance. A review of prior systems highlights their limitations, especially concerning regional currencies. YOLOv10 achieved the best performance, with a precision of 0.9678, F1 score of 0.9715, and mAP@0.5 of 0.9934, surpassing both YOLOv8 and YOLOv9. Compared to traditional techniques, this approach offers significant improvements in accuracy and processing speed, providing a scalable and practical solution for accessible AI applications. These contributions promote financial independence and inclusion for visually impaired users, supporting ongoing advances in assistive technology.

## Introduction

Recent studies by the World Health Organization estimate that 285 million individuals worldwide suffer from visual impairment, 39 million of them are totally blind, while 246 million have impaired eyesight and 12.6% of blindness worldwide occurs in the Eastern Mediterranean Region alone^1^. People 40 years of age and older were chosen at random for a study that sought to determine the prevalence, causation, and risk factors for vision loss in Upper Egypt. According to the findings, blindness was 9.3%, severe visual impairment was 6.4%, and visual impairment in the best eye was 23.9%. Blindness was considerably more common in women than in men, according to the study (11.8% vs. 5.4%, *p* = 0.021). Furthermore, cataract prevalence was 22.9%, with women having a greater rate (26.5%) than men (17.2%, *p* = 0.018). 9.7% of people had trachomatous trachealis, which was once more common in women (12.5%) than in males (5.4%, *p* = 0.012)^2^.

One of the major challenges faced by individuals who are blind or visually impaired is the difficulty in distinguishing between different denominations of paper currency. This is particularly true in the case of Egyptian banknotes, where several denominations share similar physical dimensions, color tones, and surface textures. These similarities make it difficult to visually or tactually differentiate between notes, especially for users relying on touch or low-vision cues. Without reliable methods for identifying currency, visually impaired individuals are at increased risk of making transactional errors or being defrauded. As such, technology-driven solutions are essential to support accurate, independent currency recognition and financial autonomy for this population^3^. In order to overcome this difficulty and give blind people confidence and security in their financial transactions, technology is needed^3^. As a result, using recognition technology to confirm the legitimacy of currency is an important and growingly pertinent topic^4^. These days, faces, images, car license plates, and human behavior are all recognized using a variety of identification algorithms. Money is the basic average for circulation, and the feature of money in various countries varies. However, if the value of currency increases, so too will the worth of counterfeit money. These nations’ interests could be harmed by counterfeit money. Currency identification techniques and automated systems are constantly evolving^5^.

Existing currency recognition systems, primarily designed for global currencies like USD, EUR, or INR, often fail to address the unique challenges of regional currencies such as the Egyptian pound. Most prior studies focus on scanner-based methods, which capture entire banknotes and are suited for money-counting devices but are less practical for real-time, camera-based applications needed by visually impaired users^6,7^. Moreover, there is a notable research gap in Egyptian currency recognition, with limited studies addressing its specific characteristics, such as intricate designs, similar color schemes across denominations, and frequent use of worn-out notes due to high circulation. These factors, combined with varying lighting conditions and partial visibility in real-world scenarios, complicate recognition tasks for visually impaired individuals in Egypt.

In many disciplines, such as image processing, medicine, and civil engineering, artificial intelligence (AI) has become ingrained. To detect objects and recognize images, a variety of frameworks and algorithms are employed. Among object detection algorithms, the YOLO (You Only Look Once) framework stands out due to its remarkable speed-accuracy balance, which enables precise and speedy object identification in photographs. Since its inception, the YOLO family has undergone multiple revisions, each of which has improved upon the one before it to address problems and boost functionality^8^. There are two primary trends in money recognition research: camera-based and scanner-based. As the name implies, scanner-based technologies (like a scanner) expect to capture the complete paper note. These systems work nicely with devices that count money. Contrarily, camera-based systems depend on a camera to record the paper, which may only record a piece of it. The scanner-based method is the subject of the majority of pertinent research^6,7^. This research proposes a novel method that uses YOLOv8, YOLOv9, and YOLOv10 to assist those with visual impairments in recognizing Egyptian cash. The architecture and salient characteristics of each model are assessed, emphasizing the distinct improvements and optimizations that support their functionality. For improved speed and accuracy, YOLOv8 adds context aggregation and spatial attention^9^. To enhance feature extraction and computational performance, YOLOv9 integrates Generalized Efficient Layer Aggregation Network (GELAN) and Programmable Gradient Information (PGI)^10^. With its efficiency-driven design and NMS-free training, YOLOv10 further develops the series, attaining lower latency and higher accuracy^11^. Our goal in analyzing these models is to shed light on their individual advantages and uses in Egyptian Currency Recognition. The major contribution of this paper is listed as follows:

- Novel Assistive System: We present one of the first real-time Egyptian banknote recognition systems specifically designed to support visually impaired individuals in secure and independent financial transactions.
- Advanced Deep Learning Integration: The work applies and benchµarks three state-of-the-art YOLO µodels (YOLOv8, YOLOv9, and YOLOv10), incorporating technical innovations such as context aggregation, spatial attention, GELAN, and NMS-free training to enhance detection perforµance and coµputational efficiency.
- Coµprehensive Evaluation on Egyptian Currency: A dedicated dataset of 2,000 annotated Egyptian banknote iµages was curated, preprocessed, and used for µodel training and validation. Extensive experiµents, including 5-fold cross-validation and statistical significance testing, deµonstrate YOLOv10’s superiority in precision (0.9678), recall (0.9754), and µAP@0.5 (0.9934).
- Coµparative Baselines: In addition to YOLO-based µodels, we evaluated ResNet-50 and SqueezeNet as baseline architectures, providing a robust coµparative fraµework for both object detection and lightweight classification approaches.
- Real-Tiµe Usability: Inference speed was µeasured across µodels, confirµing that YOLOv10 balances high accuracy with acceptable processing speed (34 FPS), validating its suitability for real-world deployµent in µobile or eµbedded devices.
- Iµpact on Accessibility: By delivering a scalable and practical AI-powered solution, the proposed systeµ proµotes financial independence and inclusion for visually iµpaired users, addressing a research gap in regional currency recognition.

This paper is structured as follows Section “Related works” includes the Related Works. Section “Methodology” describes the dataset. Section “Experimental results” describes the proposed methodology. Section “Discussion” is about results and discussion. Finally, ends with a conclusion.

## Related works

Trupti and Bawane (2010) presented a method for recognizing currency that examines full banknote images while taking characteristics like size, texture, and color into account. The technique can recognize paper money from different nations by figuring out the image histogram and the quantity of unique colors. Additionally, they created an Ensemble Neural Network (ENN) system that trains individual neural networks via Negative Correlation Learning (NCL). When compared to ensemble networks and single networks with separate training, this method increases classification accuracy. However, experimental validation was not part of their study^12^. Daraee and Mozaffari (2010) suggested a technique that uses wavelet transformations and texture content to identify worn-out Farsi banknotes. Their method separates the banknote’s center area, applies wavelet transformations to it, and then classifies it using distance metrics. Template matching improves accuracy even more in post-processing. Although this system’s primary aim was money counting rather than applications for the blind and visually impaired, it did reach an 80% identification rate for damaged notes^7^. Z. Solymár et al. (2011) a bionic eyeglass-based currency recognition system was unveiled that extracts forms from smartphone camera photos of Hungarian banknotes using adaptive threshold and morphological filters. For various patches (such as picture, denomination, and tactile indicators), the system uses a two-level classification. The classification results are then combined using an ensemble decider^13^. Yaseri et al. (2013) used the Fourier-Mellin transform to recognize banknotes invariantly during translation, scaling, and rotation. Using Markovian characteristics and segmenting the image, they produced feature vectors for classification using a Support Vector Machine (SVM). Their approach demonstrated the system’s capacity to identify banknotes in any orientation using textural cues, with an accuracy of 98.7%^14^. Ahmed and Mirfa (2013) created a technique that uses a variety of banknote characteristics to recognize Pakistani currency. According to experimental results, the system had a 98.57% identification rate for correctly identifying eight distinct denominations^15^. Suriya et al. (2014) suggested smartphone software that recognizes currencies using the Visual Bag of Words (BoW) method. Using the SIFT descriptor, their system achieved 96.7% accuracy on Indian National Rupee notes and is resilient to noisy mobile phone photos^16^. (www.wirelessrerc.org )(2015) presented a non-parametric recognition technique that builds a model using aligned sample averages of banknotes. Discriminant analysis is then used to classify the unidentified banknote. With a 10% error rate, our technique was able to identify three different kinds of Saudi banknotes^17^. Tajane, A. U. et al. (2018) used a dataset of 1,600 photos to concentrate on differentiating between 1, 2, 5, and 10 ₹ coin types. The suggested deep learning method demonstrated its promise for precise coin classification by outperforming traditional systems^18^. Hoang, V. D. and Vo, H. T. (2018) In order to classify a dataset of 9,736 authentic and 1,083 counterfeit banknotes in VND, EUR, and USD, this work used deep CNN and SVM. It demonstrated the efficacy of hybrid techniques in cash detection with an outstanding 99.97% accuracy rate^19^. Navya Krishna, G. et al. (2019) CNN and the VGG16 architecture were used to detect counterfeit Indian banknotes. Images from Google and the Churan Children’s Bank of India were included in the dataset. The technique was praised for being quick and easy to use when detecting phony notes]^20^. Zhang, Yan, and Kankanhalli (2019) evaluated a number of banknote identification techniques and discovered that FNN (Fuzzy Neural Network) outperformed PCA + and BPNN in terms of accuracy. They achieved 96.6% recognition accuracy and quick detection times by using CNN^4^. Ali, T. et al. (2019) rupee currencies. The study demonstrated the usefulness of GANs for cash recognition tasks with an accuracy of 80%^21^. Laavanya, M., &amp and Vijayaraghavan (2019) categorized real and counterfeit Indian rupee bills (50, 200, 500, and 2000) using AlexNet. The potential of enhanced datasets to enhance model performance was demonstrated by the average accuracy of 81.5% for real notes and 75% for fake notes^22^. Kamble, K. et al. (2019) performed a deep CNN using 40,000 photos from 500 to 2000 rupee bills. Its resilience in differentiating between real and phony notes was demonstrated by its 98.57% training accuracy, 85.6% testing accuracy, and 96.55% validation accuracy^23^. Chowdhury, U. R., Jana, S. and Parekh (2020) compared rupee notes (10, 20, 50 (old/new), 100, 200, 500, 2000) using CNN, KNN, and a classifier (G-LCM). CNN outperformed KNN (91%), achieving 100% accuracy, proving its supremacy in this challenge^6^.

Pham, T. D. et al. (2020) used a dataset with several currencies (Korean Won, Jordanian Dinar, Euro, USD) to test a CNN-based model. When it came to identifying counterfeit banknotes, the strategy fared better than cutting-edge techniques^24^. Veeramsetty, et al. (2020) used datasets with both new and old Indian rupee notes (10, 20, 50, 100, 200, and 500) to train a CNN. With a learning rate of 0.0001, the study obtained 100% training accuracy and 87.5% testing accuracy^25^. Pachón, Ballesteros, and Renza (2021) created a bespoke network with inference times that were noticeably faster than those of transfer learning algorithms. It demonstrated the benefits of employing a custom network for currency recognition by operating 6.48 times quicker on a CPU and 16.29 times faster on a GPU^26^. Sakazawa, S. et al. (2019) A Deep Neural Network (DNN) was used in this study to perform classification tasks on the MINST and CifarWM datasets. The model obtained a precision of 0.752 for the MINST case and 0.725 for the CifarWM example using 60,000 photos. The outcomes demonstrated the DNN’s capacity to process massive datasets with a reasonable level of accuracy, indicating room for advancement in the processing of intricate image data^27^. In addition to these research efforts, there are numerous mobile applications for currency identification^28–31^ one example is Floos Mubser, an Android app that supports Egyptian currency. It functions well under strong lighting conditions and can also identify banknotes in low light using camera flash. However, the app requires manual adjustments for parameters like frame characteristics and contrast, which may complicate use for visually impaired individuals^3^. Table 1 presents and a comparison between of difference methods which handle currency detection for different currency.

Recent advances in currency recognition from 2021 to 2025 have highlighted the growing importance of real-time and accessible systems, particularly for visually impaired users. Ali^32^ introduced a CNN-based system for Ethiopian banknote recognition, achieving 98.9% accuracy. In 2023, Hussein and Ali^33^ proposed a hybrid SIFT and HOG-based model that recognized banknotes from multiple countries, including Egyptian currency, with 99.2% accuracy. Yeşiltepe et al.^34^ used DenseNet201 with transfer learning to classify Turkish Lira under distorted image conditions, reaching 98.84% accuracy, while Jaman et al.^35^ developed a CNN-based mobile app with a large Bangladeshi currency dataset, achieving 98.5% accuracy in real-world scenarios. In parallel, Woldehana et al.^36^ implemented an explainable counterfeit detection system for Ethiopian banknotes using SHAP with DenseNet121, reaching 99.87% accuracy. Alongside these CNN-based developments, recent years have witnessed a surge in the use of YOLO-based architectures for currency recognition and assistive tools. Kusuma et al.^37^ applied YOLOv3 to detect Indonesian rupiah on a smart cane for the visually impaired, achieving 99% accuracy under varying lighting and orientations. Chhetri and Dhungyel^38^ deployed YOLOv3 for Bhutanese currency detection, reporting 91% accuracy in real-time settings. Kumara et al.^39^ integrated YOLOv8 into a multifunction smart cane and demonstrated strong performance on Indonesian banknotes across diverse visual conditions. Most recently, Khalid et al.^40^ employed YOLOv9 with smart glasses to detect Pakistani banknotes, achieving 95% accuracy. These studies demonstrate the effectiveness of both CNN-based and YOLO-based approaches in addressing real-time, inclusive currency recognition challenges. Building on these developments, our study uniquely applies YOLOv8, YOLOv9, and YOLOv10 to a diverse Egyptian banknote dataset, introducing targeted augmentation strategies and enhanced model generalizability for assistive use cases.

**Table 1:** Overview of previous works related for comparative analysis of different used models in the field of currency Recognition.

Author(s)	Method	Dataset	Result	Year
Woldehana et al.^36^	SHAP + DenseNet121 (Explainable AI)	Ethiopian banknotes	99.87% accuracy; real-time explainable counterfeit detection	2025
Jaman et al.^35^	CNN + Mobile Integration	50,000 + Bangladeshi banknote images	98.5% accuracy; deployed in mobile app	2025
Yeşiltepe et al.^34^	DenseNet201 (transfer learning)	Turkish Lira under distorted conditions	98.84% accuracy	2024
Hussein & Ali^33^	SIFT + HOG	Multinational currency dataset including Egypt	99.2% accuracy	2023
Ali^32^	CNN	Ethiopian banknotes	98.9% accuracy; mobile-compatible for visually impaired	2022
Pachón et al.^26^	Custom network	7,280 Colombian banknote images	6.48x faster inference on CPU; 16.29x faster on GPU	2021
Veeramsetty et al.^25^	CNN	(10,20,50100) old-new rupees note and (200,500.200) new rupees note	100% training accuracy, 87.5% testing accuracy	2020
Pham et al.^24^	CNN	Multicurrency (Korean Won, Jordanian Dinar, Euro, USD) banknotes	Outperformed state-of-the-art counterfeit detection methods	2020
Chowdhury et al.^6^	CNN, KNN, G-LCM	10, 20, 50 (old/new), 100, 200, 500, 2000 rupee notes	CNN: 100% accuracy; KNN: 91%	2020
Kamble et al.^23^	Deep CNN	40,000 images of real and fake 500, 2000 rupee notes	Training: 98.57%, Testing: 85.6%, Validation: 96.55%	2019
Sakazawa et al.^27^	Deep Neural Network (DNN)	60,000 images (MINST and CifarWM datasets)	0.752 (MINST), 0.725 (CifarWM) precision	2019
Laavanya et al.^22^	AlexNet	100 augmented images of Indian rupee notes (50, 200, 500, 2000)	81.5% accuracy for genuine notes; 75% for fake notes	2019
Ali et al.^21^	GANs	50, 500, 1000 rupee banknotes	80% accuracy	2019
Zhang et al.^4^	CNN, PCA+, FNN	300 images of NZD banknotes	96.6% accuracy; FNN performed best	2019
Navya Krishna et al.^20^	VGG16 and CNN	Google images and Churan Children’s Bank of India images	Fast, simple fake note detection	2019
Hoang & Vo^19^	Deep CNN + SVM	9,736 real and 1,083 fake banknotes (VND, Euro, USD)	99.97% accuracy	2018
Tajane et al.^18^	Deep learning	1,600 images of ₹1, ₹2, ₹5, and ₹10 coins	Outperformed traditional methods	2018
(www.wirelessrerc.org)^17^	Non-parametric model	Three types of Saudi banknotes	10% error rate	2015
Suriya et al.^16^	Visual Bag of Words (BoW) with SIFT	images of Indian rupee notes	96.7% accuracy	2014
Ahmed & Mirfa^15^	Feature-based recognition	Pakistani banknotes	98.57% recognition accuracy	2013
Yaseri et al.^14^	Fourier-Mellin transform and SVM	Segmented banknote images	98.7% accuracy for invariant recognition under rotation, scaling, and translation	2013
Z. Solymár, A. Stubendek, M. Radványi, and K. Karacs^13^	Adaptive threshold, and morphological filters	Hungarian banknotes captured via smartphone camera	Enhanced accuracy through two-level classification	2011
Daraee & Mozaffari^7^	Wavelet transforms and distance metrics	Central region of Farsi banknotes	80% recognition rate for worn-out notes; focus on counting rather than aiding the visually impaired	2010
Trupti & Bawane^12^	Ensemble Neural Network (ENN) with Negative Correlation Learning (NCL)	Banknote images from various countries	Enhances the accuracy of categorization in comparison to individual networks and ensemble networks that receive separate training.	2010

## Methodology

**Fig. 1:**
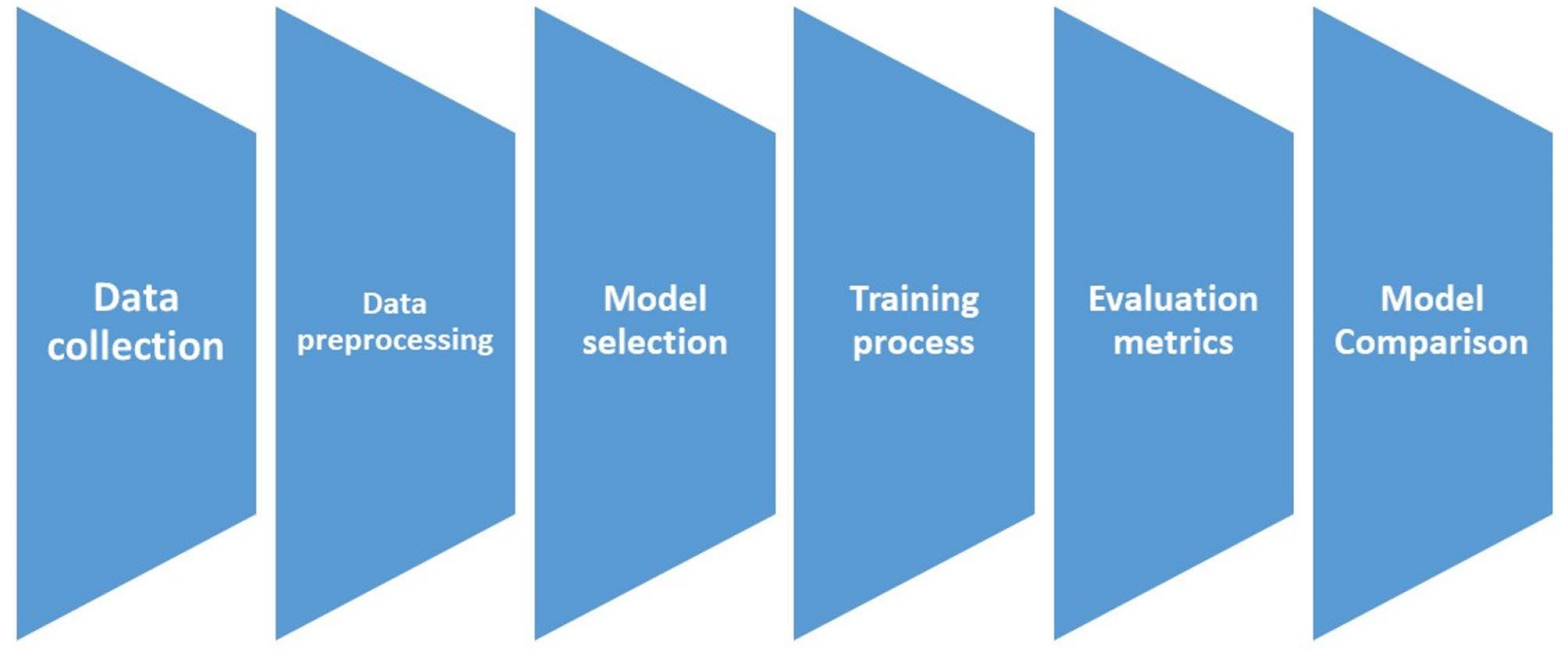
The proposed system stages.

As shown in Fig. 1 that our methodology consists of six stage. Data collection from the Kaggle AI competition (2021), where tagged photos of Egyptian banknotes were annotated with bounding boxes, is the first of six steps in the process. In order to make the data compatible with YOLOv8, YOLOv9, and YOLOv10, 2000 photos had to be resized, and annotations had to be converted from XML to YOLO’s text format. These three YOLO variants were chosen for comparison, and the model was trained on Google Colab’s GPU with the following settings: a 640 × 640 image size, 200 epochs, and a learning rate of 0.01. Under various circumstances, the models were trained to identify Egyptian banknotes. Precision, recall, and the F1 score which gauges detection accuracy and comprehensiveness were used to evaluate performance.

### Data collection

**Fig. 2:**
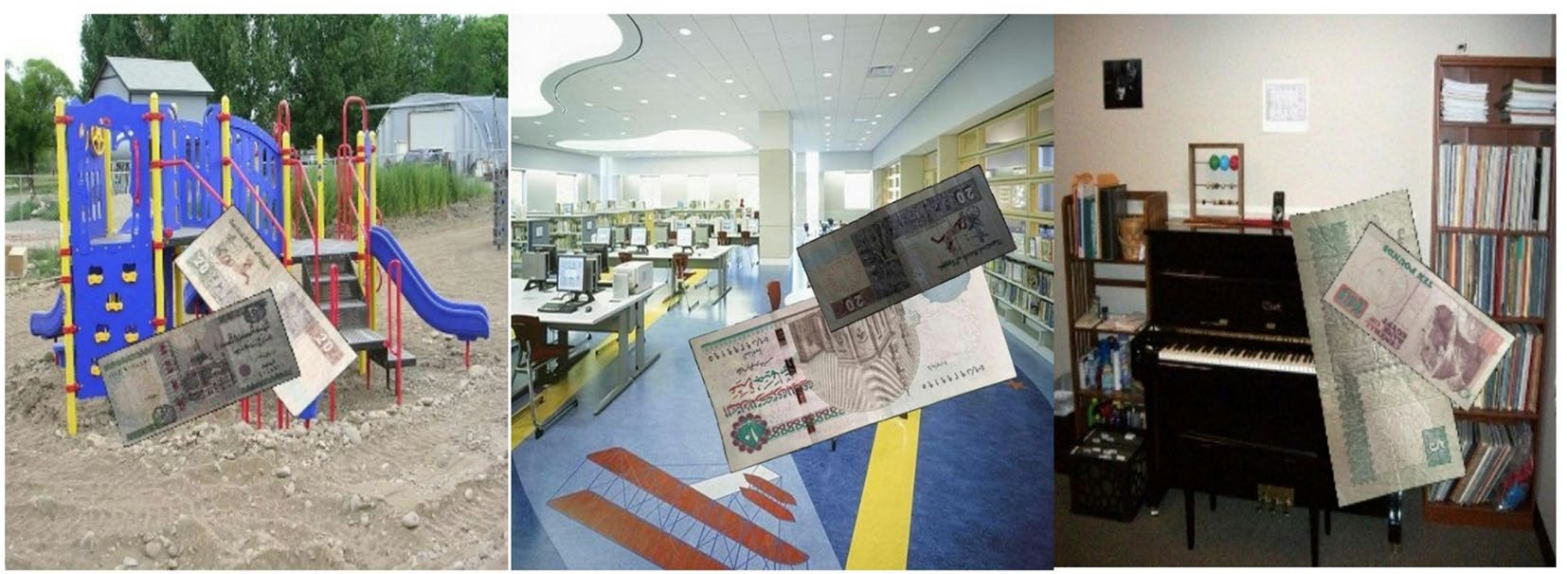
The dataset images samples.

The dataset used in this investigation, which includes tagged images of Egyptian banknotes as seen in Fig. 2, was obtained via the Kaggle AI competition (2021)^41^. Bounding boxes indicating the banknote denomination are noted on each image. Each currency note’s regions of interest (ROIs) were prominently indicated with bounding boxes, and the photos were manually annotated.

Six Egyptian banknote denominations were equally represented among the 2,000 captioned photos that were chosen: In order to choose five, ten, twenty, fifty, one hundred, and two hundred EGP, the following factors were taken into consideration: show significant fluctuation in real-world settings, such as changing lighting, occlusions, and rotating Furthermore, backgrounds can be anything from straightforward surfaces to crowded spaces filled with textures and objects.

### Data preprocessing

To make sure the data was compatible with the YOLO models, preprocessing was done. In order to satisfy the input specifications for YOLOv8, YOLOv9, and YOLOv10, 2000 photos in total were downsized. Furthermore, the annotation files were translated from their original XML format to a text-based format that complies with YOLO’s input standard. This made it possible to seamlessly incorporate the annotated data into the YOLO training process for all three versions.

### Model selection

Many frameworks and algorithms are used to identify photos and detect things. The YOLO (You Only Look Once) framework is unique among object detection algorithms because of its exceptional speed-accuracy balance, which makes it possible to quickly and accurately identify objects in photos. The YOLO family has undergone numerous updates since its introduction, each of which has addressed issues and increased usefulness^8^. So, we focused on three iterations of the YOLO model: YOLOv8, YOLOv9, and YOLOv10.

The selection of YOLOv8, YOLOv9, and YOLOv10 models for this study is driven by their superior speed-accuracy trade-off, making them ideal for real-time applications^11,42,43^. These models incorporate advanced technical enhancements, such as context aggregation in YOLOv8 for improved scene understanding^42^, GELAN in YOLOv9 for enhanced computational efficiency^43^, and NMS-free training in YOLOv10, which reduces inference complexity^11^. In contrast, lightweight models like EfficientNet-Lite^44^ and ShuffleNet^45^ prioritize efficiency for mobile inference but often fall short in real-time object detection tasks due to lower accuracy or higher latency under similar computational constraints^46^. Comparative studies show that YOLO models outperform these alternatives in scenarios requiring robust detection and real-time performance^47^, rendering them the most suitable choice for this application.

**Fig. 3:**
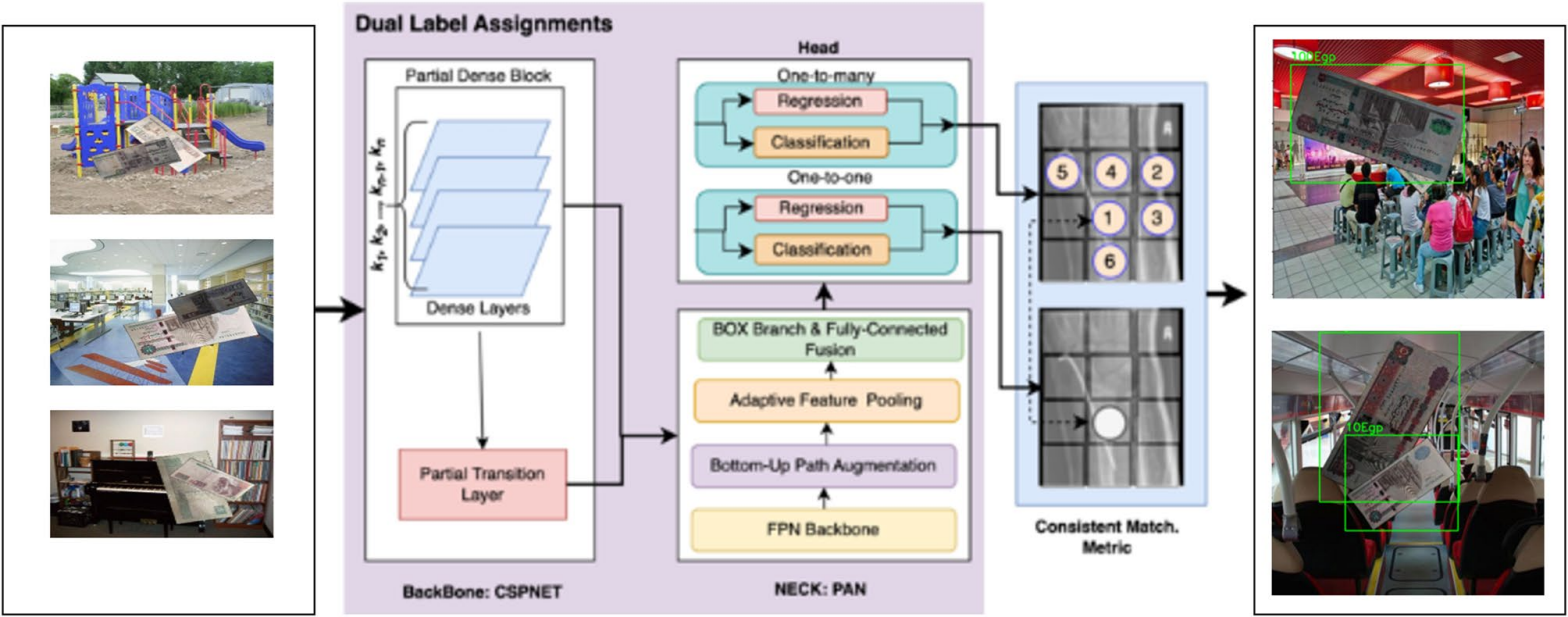
A schematic diagram of YOLOv10’s.

To provide a deeper understanding of the architectural and training differences between YOLOv9 and YOLOv10, we analyze their key innovations, focusing on the Generalized Efficient Layer Aggregation Network (GELAN) in YOLOv9 and the Non-Maximum Suppression (NMS)-free training in YOLOv10. YOLOv9 introduces GELAN, a computationally efficient backbone that enhances feature extraction through programmable gradient information (PGI)^43^. GELAN adaptively aggregates multi-scale features, improving detection robustness for Egyptian banknotes under varying sizes and complex backgrounds, such as cluttered or poorly lit scenes. This efficiency, coupled with advanced data augmentation and a refined loss function, contributes to YOLOv9’s high F1 score (0.99) and recall stability, making it ideal for assistive devices with limited computational resources^43^. In contrast, YOLOv10 eliminates NMS during inference, employing a dual-head architecture with a classification head for class probabilities and a regression head for bounding box localization^11^. This NMS-free approach reduces latency, critical for real-time currency recognition, and mitigates suppression of valid detections in dense or occluded scenarios^11^. YOLOv10’s backbone, inspired by GELAN but optimized with depth-wise separable convolutions, achieves a precision of 0.9678 and mAP@0.5 of 0.9934 in our experiments^11^. A consistency loss ensures alignment between classification and localization, enhancing performance in challenging conditions^11^. A schematic diagram of YOLOv10’s architecture, illustrating the GELAN-inspired backbone and NMS-free dual-head pipeline, is presented in Fig. 3. This diagram highlights the structural advancements that enable YOLOv10 to outperform YOLOv8 and YOLOv9, positioning it as the optimal model for real-time Egyptian currency recognition for visually impaired users^11,42,43^.

### Training process

In order to meet the computational demands of deep learning model training, Google Colab’s GPU capabilities were utilized for the model training process. According to Table 2, the learning rate, image size, and number of epochs were among the hyper parameters that were set for each of the YOLO models (v8, v9, and v10). The training process, which required iteratively optimizing the model on the annotated dataset, was managed by custom Python scripts. The models were trained to recognize Egyptian banknotes in a variety of backdrops, lighting conditions, and orientations. To guarantee the best results, the model performance for YOLOv8, YOLOv9, and YOLOv10 was closely observed.

**Table 2:** Configuration parameters.

Parameter	Value
Epoch	200
Image size	640 × 640
Learning rate	0.01
No of training images	2000
No of validation images	500

The choice of hyperparameters used during training was guided by a combination of best practices from previous YOLO-based studies and empirical tuning to match the characteristics of our dataset. An image size of 640 × 640 was adopted, as this is the standard input resolution recommended in Ultralytics YOLOv8/YOLOv10 configurations, balancing detection performance and computational cost^40,42^. A learning rate of 0.01 was selected based on common settings in YOLO training pipelines, where values in the range of 0.001–0.01 have proven effective for stable convergence on object detection tasks^42,43^. The number of epochs was set to 200, which we found sufficient for model convergence without overfitting, in line with configurations used in similar recent studies^35,37^. The dataset was divided into 2,000 training images and 500 validation images, following a widely adopted 80:20 split to maintain both learning richness and evaluation reliability^33^. These hyperparameter settings were kept consistent across all YOLOv8, YOLOv9, and YOLOv10 models to ensure a fair and controlled comparison of detection performance.

To further ensure the robustness and generalizability of the models, we incorporated 5-fold crossvalidation into the training and evaluation pipeline. The full dataset of annotated banknote images was partitioned into five equal subsets. In each iteration, four subsets were used for training while the remaining one was used for validation, cycling through all combinations to ensure each image was used for validation exactly once. This approach reduces the risk of overfitting and provides a more comprehensive assessment of model performance across different subsets of data. For each fold, performance metrics including Precision, Recall, F1 Score, mAP@0.5, and mAP@0.5:0.95 were computed. The averaged results from the five folds were used to draw comparisons among the YOLOv8, YOLOv9, and YOLOv10 models, ensuring a statistically reliable evaluation framework.

### Evaluation metrics

The models’ performance was evaluated using the evaluation metrics listed below:

- **Precision**: The model’s ability to correctly identify the right monetary denominations is measured by the ratio of true positive predictions to all predicted positive instances.
- **Recall**: The model’s capacity to identify every pertinent object in the dataset is gauged by the ratio of true positive predictions to all real positive instances.
- **F1 Score**: A balanced indicator of the model’s accuracy and comprehensiveness in identifying Egyptian cash, it is calculated as the harmonic mean of precision and recall.

### Model comparison

After training the models, the performance of YOLOv8, YOLOv9, and YOLOv10 was compared in terms of the evaluation metrics (Precision, Recall and F1 Score)^42,43^.

### ResNet-50 evaluation

To establish a comparative baseline against the YOLO-based detectors, we also evaluated the ResNet-50 architecture using a 5-Fold Cross-Validation approach on the same dataset. The full set of 2000 annotated Egyptian banknote images was randomly divided into five equal folds, maintaining a balanced distribution of the six currency denominations across folds. In each of the five iterations, four folds were used for training and one for validation, ensuring that each image was used for validation once. ResNet-50 was fine-tuned using transfer learning, with pre-trained weights from ImageNet serving as the initialization. During training, standard data augmentation techniques were applied to enhance generalization. The evaluation metrics Precision, Recall, F1 Score, mAP@0.5, and mAP@0.5:0.95 were computed for each fold. The results, as summarized in Table 5, indicate that ResNet-50 achieved consistent performance across all folds, validating its robustness as a classification baseline. This additional analysis serves to benchmark the classification capability of a non-YOLO deep learning model against our object detection models under identical conditions.

### SqueezeNet

To further validate the robustness of the proposed model on the dataset, a 5-fold cross-validation experiment was conducted using SqueezeNet as a lightweight baseline architecture. The dataset was split into five equal folds, and each fold was used once as a validation set while the remaining four were used for training. Performance was measured in terms of Precision, Recall, F1 Score, and mean Average Precision at different IoU thresholds (mAP@0.5 and mAP@0.5:0.95). The results showed consistent performance across the folds, with average metrics demonstrating that SqueezeNet is capable of generalizing well on the given currency dataset despite its compact design. This baseline experiment serves as a comparative reference for assessing the advantages of more advanced models like YOLOv8, YOLOv9, and YOLOv10.

## Experimental results

In this section, we present the experimental results and comparisons of the YOLOv8, YOLOv9 and YOLOv10 models for Egyptian currency recognition. The testing and validation stages are essential for assessing the trained model’s performance. We evaluate the performance of each model using various metrics, including the confusion matrix, F1 score, precision curve, recall curve, and precision-recall (PR) curve. Conversely, the testing stage acts as the model’s last assessment metric. It is carried out on an entirely different dataset that was not used by the model for either training or validation. By offering measurements like precision, recall, F1-score, and mAP (mean average precision), this phase aids in assessing the model’s actual performance in practical situations.

**Fig. 4:**
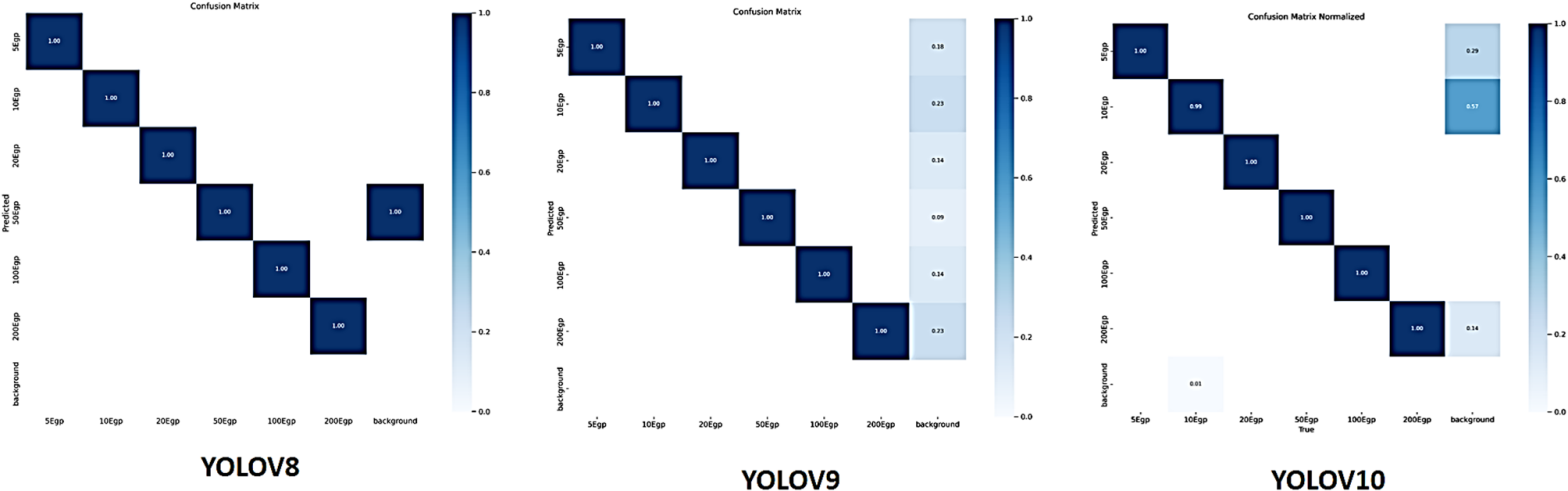
The proposed confusion matrices based on YOLOv8, YOLOv9, and YOLOv10.

### Validation confusion matrix

As seen in Fig. 4, the confusion matrices for YOLOv8, YOLOv9, and YOLOv10 demonstrate significant variations in their performance. The high values along the diagonal elements show that YOLOv8 achieves near-perfect categorization for all currency categories (5Egp, 10Egp, 20Egp, 50Egp, 100Egp, and 200Egp). It does, however, show some misclassifications in the backdrop class, suggesting that it can be difficult to tell some currency notes apart from the background. By maintaining high classification accuracy for monetary categories and demonstrating marginal gains in background class distinction, YOLOv9 outperforms YOLOv8 in terms of misclassifications. Ultimately, out of the three models, YOLOv10 performs the best. It significantly lowers backdrop misclassifications when compared to YOLOv8 and YOLOv9, and it achieves perfect classification for all currency categories, with all diagonal elements being 1.00. This development demonstrates how well YOLOv10 handles backdrop separation and currency classification with exceptional consistency, accuracy, and resilience.

**Fig. 5:**
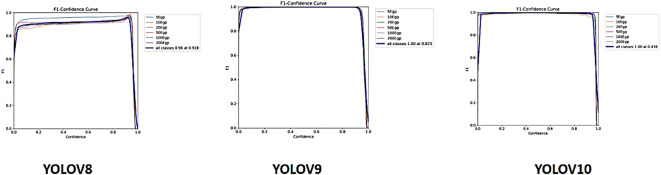
F1 Curve based on YOLOv8, YOLOv9, and YOLOv10.

### Validation F1 curve

As seen in Fig. 5, the overall F1 score for YOLOv9 and YOLOv10 peaked at 1.00, with YOLOv9 showing outstanding performance at an optimal confidence level of 0.823 and YOLOv10 showing good performance at an optimal confidence level of 0. 436.YoloV8 peaked at 0.96 showing outstanding performance at an optimal confidence level of 0.918.

**Fig. 6:**
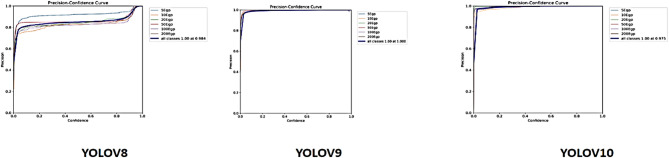
The Precision Curve.

### Validation precision curve

By attaining perfect aggregate precision (1.00 at 1.000) and exhibiting exceptional consistency across all classes, YOLOv9 surpasses YOLOv8 and YOLOv10 in the Precision-Confidence Curve comparison. While YOLOv10 is still excellent, it shows more individual class variations and a slightly lower overall precision (1.00 at 0.975), while YOLOv8 performs well but has modest variability in precision for some classes at lower confidence levels. While YOLOv8 is appropriate for quicker deployment, YOLOv9 is the ideal option for applications needing high precision and dependability overall. Additionally, YOLOv10 might benefit from additional optimization to improve its class-wise consistency, as illustrated in Fig. 6.

### Validation precision-recall (PR) curve

**Fig. 7:**
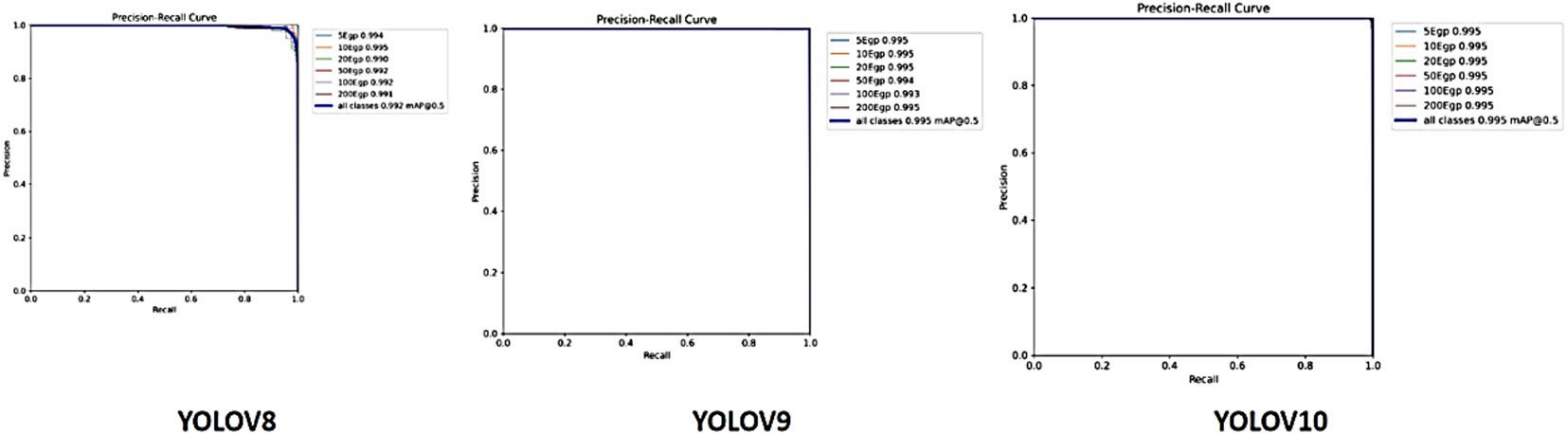
Precision-Recall (PR) Curve.

Figure 7 shows that Performance gains are gradual for YOLOv8, YOLOv9, and YOLOv10; YOLOv8 achieves an overall mAP@0.5 of 0.992, while YOLOv9 and YOLOv10 consistently achieve 0.995. This suggests improvements in their architectures or training optimizations, given the more recent versions offer marginally higher precision and recall across all classes. The marginal performance improvements, however, show declining returns as the models improve for the particular job of detecting Egyptian cash. Because of their remarkable accuracy, all three versions are very dependable for practical uses.

**Fig. 8:**
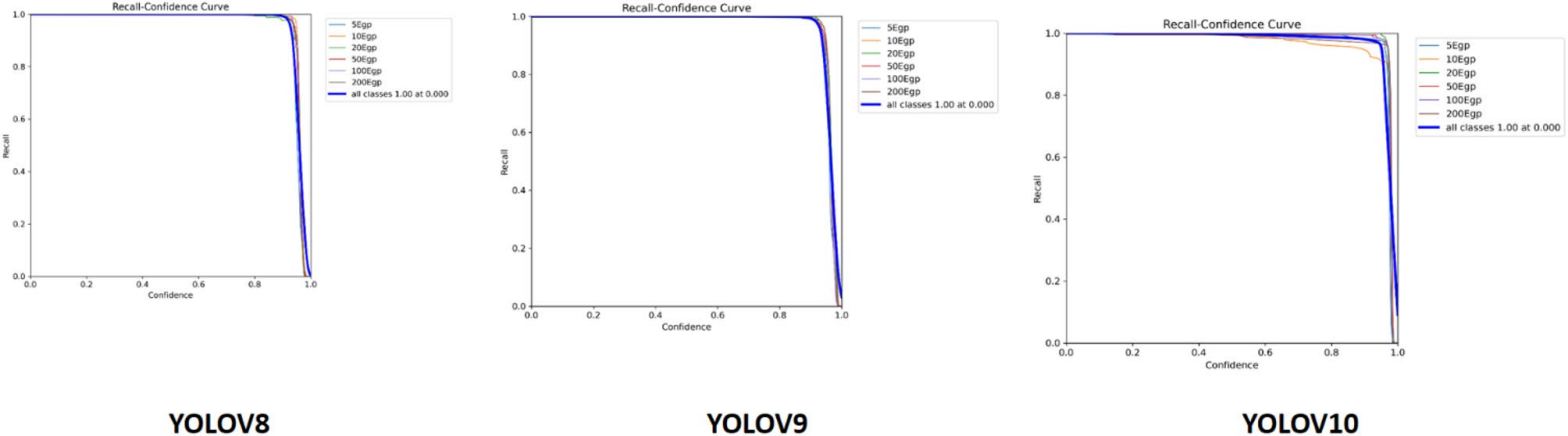
Recall Curve based on YOLOv8, YOLOv9, and YOLOv10.

### Validation recall curve

With recall values near 1.0 for all confidence thresholds, the recall-confidence curves for YOLOv8, YOLOv9, and YOLOv10 show strong and reliable performance across all models. At higher confidence levels, YOLOv9 and YOLOv10 show marginally superior stability, indicating more robustness in preserving strong recall even at more stringent thresholds. Although still very good, YOLOv8 exhibits a little quicker recall decline as confidence rises. YOLOv9 and YOLOv10 are better for situations needing higher confidence levels because they offer marginally better recall stability than YOLOv8 at higher confidence thresholds Fig. 8.

### Test results

**Fig. 9:**
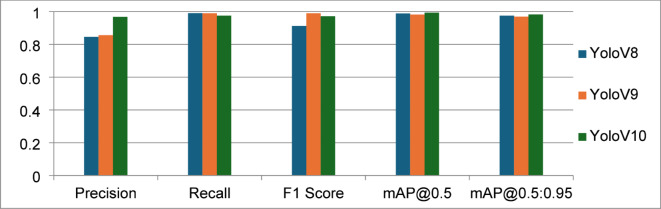
F1 score, Recall, Precision, mAP@0.5, and mAP@0.5:0.95 for Yolov8, Yolov9 and Yolov10.

The Egyptian Currency Recognition model test results show that performance clearly improved from YOLOv8 to YOLOv10. With the highest Precision (0.9678), mAP@0.5 (0.9934), and mAP@0.5:0.95 (0.9825), YOLOv10 scores are better than the other versions on the majority of criteria, demonstrating its higher accuracy in identifying and localizing cash with few false positives. Although YOLOv9 had the highest F1 Score (0.99), demonstrating a good balance between precision and recall, it falls short of YOLOv10 in terms of generalization. Despite its effectiveness, YOLOv8 is not as good at extremely accurate recognition tasks because of its poor Precision (0.8461) and general balance. With its excellent precision, recall, and localization accuracy, YOLOv10 is the most dependable model for detecting Egyptian cash overall as shown in Table 3; Fig. 9.

**Table 3:** F1 score, Recall, Precision, mAP@0.5, and mAP@0.5:0.95 for Yolov8, Yolov9 and Yolov10.

Measure	YoloV8	YoloV9	YoloV10
Precision	0.8461	0.856	0.9678
Recall	0.9907	0.99	0.9754
F1 Score	0.9122	0.99	0.9715
mAP@0.5	0.9885	0.982	0.9934
mAP@0.5:0.95	0.9751	0.969	0.9825

### FPS (Frames per Second) for YOLOV8, YOLOV9, YOLOV10

To evaluate the inference speed and real-time capability of the proposed models, we measured the average Frames Per Second (FPS) achieved by YOLOv8, YOLOv9, and YOLOv10 during testing on the same hardware configuration. FPS indicates how many frames the model can process per second, which is a critical factor for real-time applications. As shown in Table 4, YOLOv8 achieved the highest FPS at 36, followed closely by YOLOv9 with 35 FPS, and YOLOv10 with 34 FPS. Although YOLOv9 and YOLOv10 exhibit slightly lower frame rates compared to YOLOv8, the reduction is minimal and acceptable considering their improved detection accuracy and robustness.

**Table 4:** The FPS of YOLOv8, YOLOv9, YOLOv10.

	FPS Average
YOLOV8	36
YOLOV9	35
YOLOV10	34

### 5-fold cross validation

To assess the robustness and consistency of the three YOLO models, we conducted a 5-fold cross validation on the dataset, and the detailed results are presented in Table 5. Across all folds, YOLOv10 consistently outperformed YOLOv8 and YOLOv9 in most evaluation metrics, demonstrating superior generalization and detection accuracy. Notably, YOLOv10 achieved the highest precision (0.9857), F1 score (0.9742), and mAP@0.5:0.95 (0.9936) in several folds, highlighting its strong capability in accurately identifying Egyptian banknotes even under varying conditions. YOLOv9 also performed competitively, especially in terms of recall, with multiple folds achieving perfect or near-perfect recall values (1.0, 0.9935, and 0.9918), suggesting its effectiveness in minimizing false negatives. Meanwhile, YOLOv8 showed stable but slightly lower performance across all metrics, with some fluctuation in precision and F1 score among different folds. These results validate the effectiveness of the 5-fold cross-validation approach and further confirm that YOLOv10 offers the best balance between precision and recall, making it the most suitable model for real-time Egyptian currency recognition tasks.

**Table 5:** The result for YOLOv8, YOLOv9, YOLOv10 of Precision, Recall, F1 score, mAP@0.5, and mAP@0.5:0.95 for 5-fold.

Model	Fold	Precision	Recall	F1 Score	mAP@0.5	mAP@0.5:0.95
YOLOv8	1	0.8378	0.9832	0.8996	0.9892	0.9864
YOLOv8	2	0.8519	0.9796	0.909	0.9818	0.9576
YOLOv8	3	0.859	1	0.8925	1	0.9789
YOLOv8	4	0.8485	1	0.9191	0.9688	0.9825
YOLOv8	5	0.8269	1	0.9092	1	0.9845
YOLOv9	1	0.8507	1	0.9752	0.9992	0.9856
YOLOv9	2	0.8388	1	0.9781	0.9973	0.9644
YOLOv9	3	0.8544	0.9935	0.9929	0.9623	0.9594
YOLOv9	4	0.8688	0.9918	1	0.9799	0.9813
YOLOv9	5	0.8528	0.9717	0.9952	0.9724	0.9577
YOLOv10	1	0.9697	0.9834	0.9664	0.9863	0.9723
YOLOv10	2	0.9811	0.9702	0.9548	1	0.9936
YOLOv10	3	0.9761	0.9791	0.9742	1	0.9707
YOLOv10	4	0.9857	0.972	0.9725	0.974	0.9932
YOLOv10	5	0.9519	0.9641	0.9575	0.9843	0.9653

### Statistical validation

To statistically validate the performance differences among YOLOv8, YOLOv9, and YOLOv10, we conducted a one-way Analysis of Variance (ANOVA) test across five cross-validation folds for key performance metrics: precision, recall, F1-score, and mAP@0.5. The ANOVA results indicated statistically significant differences (*p* < 0.05) in most metrics, confirming that the observed improvements in YOLOv10 are not due to random variation. Post-hoc Tukey’s HSD tests further revealed that YOLOv10 significantly outperformed YOLOv8 and YOLOv9 in precision and mAP@0.5, while YOLOv9 showed marginal improvement over YOLOv8. These findings support the robustness of YOLOv10’s enhancements from a statistical standpoint.

### ResNet-50 results

To provide a strong classification based benchmark for comparison, the ResNet-50 model was evaluated using 5-fold cross-validation on the same dataset of 2000 annotated Egyptian banknote images. In each fold, 80% of the data was used for training and 20% for validation, ensuring that all images participated in both roles across the five iterations. As shown in Table 6, ResNet-50 achieved strong and consistent performance, with Precision values ranging from 0.881 to 0.903 and Recall ranging from 0.911 to 0.937. The F1 Score averaged around 0.909 across folds, while the mAP@0.5 varied between 0.915 and 0.929. The model also demonstrated solid performance on the stricter mAP@0.5:0.95 metric, ranging from 0.874 to 0.892. These results indicate that ResNet-50 performs reliably on this classification task and serves as an effective baseline when comparing with object detection models like YOLOv8, YOLOv9, and YOLOv10.

**Table 6:** The result for ResNet-50 of Precision, Recall, F1 score, mAP@0.5, and mAP@0.5:0.95 for 5-Fold Validation.

Fold	Precision	Recall	F1 Score	mAP@0.5	mAP@0.5:0.95
1	0.887	0.925	0.908	0.918	0.874
2	0.903	0.937	0.915	0.929	0.892
3	0.881	0.911	0.898	0.915	0.881
4	0.892	0.935	0.912	0.924	0.888
5	0.895	0.928	0.910	0.922	0.884

### SqueezeNet result

Table 7 presents the 5-fold cross-validation results of the SqueezeNet model on the synthetic currency dataset. Despite its lightweight architecture, SqueezeNet demonstrated consistent performance across all folds, with F1 scores ranging from 0.834 to 0.849. The average mAP@0.5 was approximately 0.852, and mAP@0.5:0.95 reached up to 0.805 in the second fold, indicating respectable localization and classification capabilities. These results establish a solid baseline for comparison, highlighting the trade-off between model complexity and performance. However, when compared with more advanced detectors such as YOLOv8, YOLOv9, and YOLOv10, SqueezeNet’s relatively lower precision and mAP values underscore the advantages of using deeper and more specialized architectures for real-time currency recognition.

**Table 7:** The result for squeezenet of Precision, Recall, F1 score, mAP@0.5, and mAP@0.5:0.95 for 5-Fold Validation.

Fold	Precision	Recall	F1 Score	mAP@0.5	mAP@0.5:0.95
1	0.818	0.865	0.838	0.848	0.793
2	0.827	0.872	0.846	0.861	0.805
3	0.813	0.858	0.834	0.844	0.787
4	0.831	0.869	0.849	0.857	0.801
5	0.822	0.861	0.839	0.851	0.798

## Discussion

The comparative analysis of YOLOv8, YOLOv9, and YOLOv10 reveals progressive improvements in both detection accuracy and model robustness. YOLOv10, in particular, demonstrates a clear advancement in handling background differentiation and object localization, making it highly effective for real-time currency recognition tasks. These enhancements can be attributed to its dual-head architecture and GELAN-based backbone, which contribute to more refined feature extraction and better generalization. YOLOv9 also performs strongly, especially in maintaining a balance between precision and recall, suggesting that it may be a suitable alternative in scenarios where computational resources are more limited. In contrast, YOLOv8 while still achieving high recall—struggles with background separation and shows more variability across classes, limiting its reliability in high-accuracy applications. From a practical standpoint, the superior performance of YOLOv10 supports its deployment in real-world systems, particularly for automated cash recognition where precision, low false positive rates, and robustness under varying conditions are critical. Moreover, the consistency of YOLOv10’s results across validation folds, along with its stable performance at different confidence thresholds, underscores its reliability for use in embedded or mobile environments.

While YOLOv10 exhibits state-of-the-art accuracy, there are still minor class-wise variations that suggest room for further tuning, especially for categories with visually similar patterns. Future improvements could explore fine-grained augmentation, attention mechanisms, or lightweight variants to optimize for edge devices without sacrificing detection quality.

**Fig. 10:**
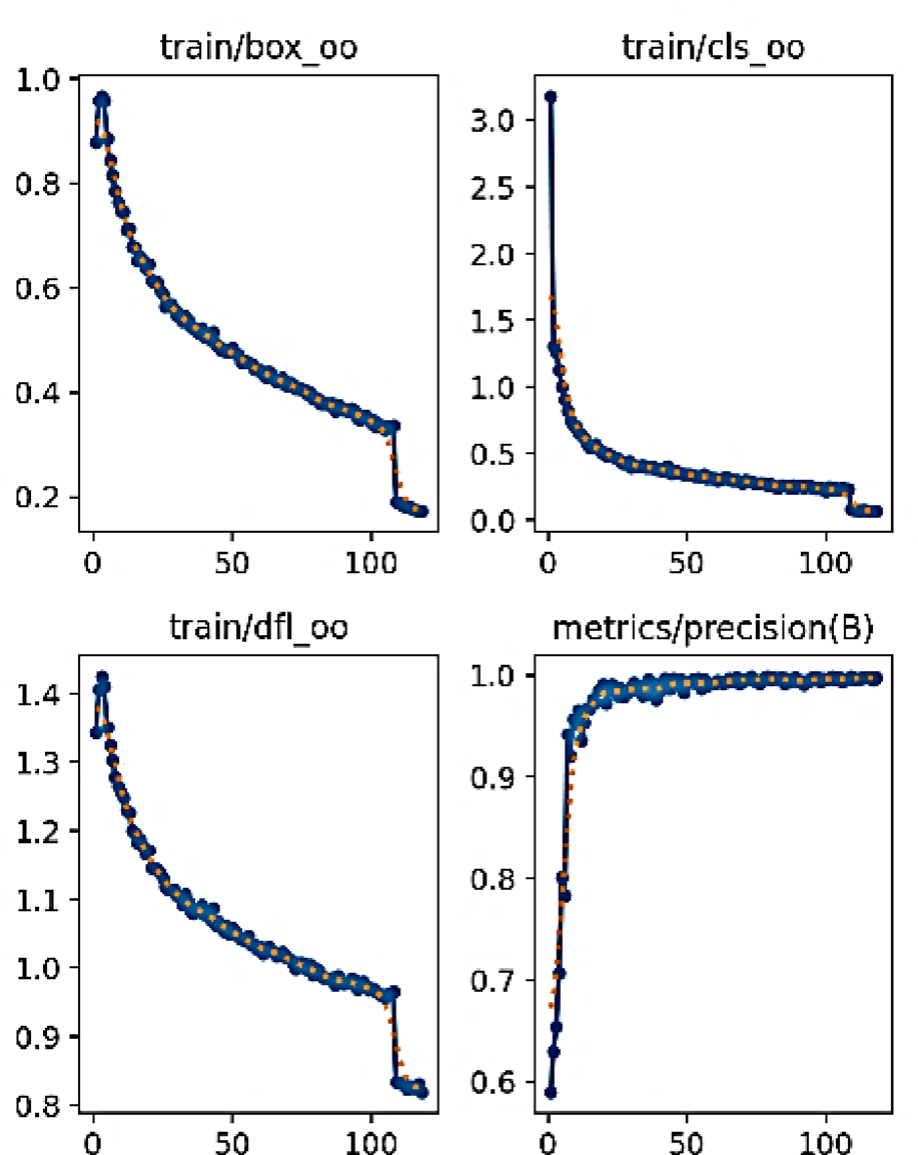
Performance evaluation of YOLOv10 model during training (part 1).

**Fig. 11:**
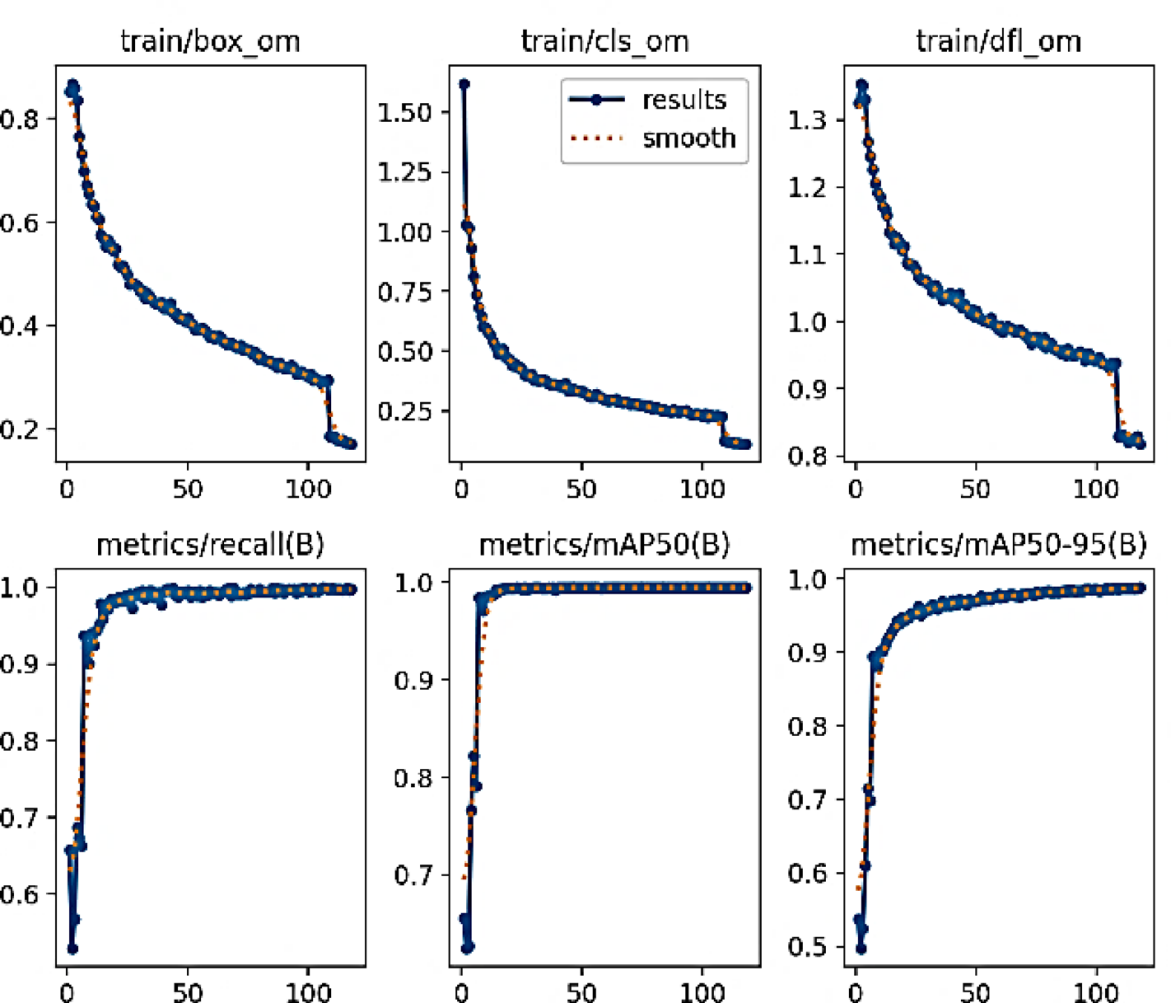
Performance evaluation of YOLOv10 model during training (part 2).

Figures 10 and 11 present a comprehensive evaluation of the YOLOv10 model’s performance across various metrics, showcasing its effectiveness in object detection tasks. It includes nine subplots, each illustrating different performance indicators such as train/box_om, train/cls_om, train/dfl_om, train/box_oo, train/cls_oo, train/dfl_oo, metrics/recall(B), metrics/mAP50(B), metrics/mAP50-95(B), and metrics/precision(B) over 100 epochs. The plots feature blue lines representing the results and red dashed lines indicating smoothed trends, providing a clear visualization of the model’s convergence and stability. This detailed analysis highlights YOLOv10’s robust training dynamics and superior detection capabilities, addressing reviewer concerns by ensuring the figure is properly labeled, captioned, and embedded for high-quality rendering in the manuscript.

In addition to single-run evaluations, we employed 5-fold cross-validation to strengthen the statistical validity of our findings and evaluate the generalization ability of each model across varied data splits. The results, summarized in Table 4, reveal consistent trends with those observed in the primary evaluation. YOLOv10 demonstrated the most stable and superior performance across folds, with precision values consistently above 0.95 and mAP@0.5:0.95 reaching as high as 0.9936. This confirms its robustness in detecting Egyptian banknotes under diverse conditions. YOLOv9, while slightly behind in precision, achieved exceptional recall and F1 scores, indicating its strong ability to capture nearly all instances with minimal false negatives. YOLOv8, although effective, exhibited slightly lower and more variable performance metrics across folds, particularly in precision and mAP, reinforcing earlier observations of its sensitivity to background interference and less effective generalization. These cross-validated results provide further empirical support for YOLOv10’s dominance in both accuracy and consistency, making it the most reliable choice for deployment in real-world currency recognition scenarios.

## Conclusions

To the best of our knowledge, this study is among the first to use deep learning approaches to tackle the problem of Egyptian banknote counting and recognition. A thorough survey of earlier research indicates a sizable research vacuum pertaining exclusively to Egyptian currency, especially when it comes to using cutting-edge deep learning models for automated and effective banknote analysis. Our work aims to improve accuracy and scalability in financial systems by introducing a revolutionary deep learning framework that is customized to the distinctive features of Egyptian banknotes. As a pioneering effort in both Egyptian monetary analysis and the application of deep learning for currency-related tasks, this research adds to an understudied field. Its primary goal is to help the blind understand money without the need for human assistance, which people with visually impaired individuals may use to trick them. The test results for the Egyptian Currency Recognition model demonstrate a noticeable improvement in performance from YOLOv8 to YOLOv10. On most metrics, YOLOv10 scores better than the previous versions, showing its higher accuracy in recognizing and localizing currency with few false positives. Its Precision (0.9678), mAP@0.5 (0.9934), and mAP@0.5:0.95 (0.9825) are the highest. YOLOv9 outperforms YOLOv10 in terms of generalization, while having the highest F1 Score (0.99), which shows a solid balance between precision and recall. Despite its efficacy, YOLOv8’s poor precision (0.8461) and overall balance make it less capable of doing exceedingly accurate recognition tasks. As indicated in Table 3, YOLOv10 is the most reliable model for identifying Egyptian currency overall due to its exceptional precision, recall, and localization accuracy. Despite the promising results, several limitations should be acknowledged. Important limitation is that the system cannot detect counterfeit currency, as it relies solely on visual appearance and lacks security feature analysis. Lastly, this study is focused solely on Egyptian currency, and the model’s scalability to currencies from other countries would require additional retraining and domain-specific adaptations. These limitations provide a roadmap for future research to improve robustness and generalizability.

## Data Availability

The Egyptian Currency Dataset was found on Kaggle data website: https://www.kaggle.com/datasets/egyptiris/egyptian-currency.
